# Impact of Well-being Interventions for Siblings of Children and Young People with a Chronic Physical or Mental Health Condition: A Systematic Review and Meta-Analysis

**DOI:** 10.1007/s10567-018-0253-x

**Published:** 2018-02-15

**Authors:** Mhairi McKenzie Smith, Snehal Pinto Pereira, Lynette Chan, Charlotte Rose, Roz Shafran

**Affiliations:** UCL Great Ormond Street Institute of Child Health, 30 Guilford Street, London, WC1N 1EH UK

**Keywords:** Siblings, Chronic, Psychological, Intervention

## Abstract

Siblings of children and young people with a chronic illness are at increased risk of poor psychological functioning. A number of studies have attempted to implement and evaluate interventions targeting the psychological well-being of this at-risk group. This systematic review summarises the evidence regarding psychological functioning of siblings following an intervention targeting their well-being. The meta-analysis considered behaviour and knowledge, two of the most frequently studied outcomes. The following databases were used: PsycINFO, EMBASE, CINAHL, PubMed, Scopus and Web of Science. Seventeen studies were eligible to be included in the systematic review and eight in the meta-analysis. Results from the systematic review reflected the inconsistency of intervention evaluations in this area with a high level of heterogeneity and a total of 23 outcomes considered across the 17 included studies. The meta-analysis estimated effect sizes using a standardised mean difference (SMD) approach. Pre-post analysis suggested significant improvement in behavioural outcomes and knowledge of their sibling’s health conditions with a SMD of − 0.44 [95% CI (− 0.6, − 0.29); *p* = 0.000] and 0.69 [(95% CI = 0.42, 0.96); *p* = 0.000], respectively. The SMD was not significant for behavioural outcomes when considering treatment–control studies. In conclusion, the findings suggest interventions for well-being have a positive effect on the psychological functioning of siblings of children and young people with a chronic illness, but their specificity needs to be established. There is a need for further, more methodologically robust research in this area.

**Electronic supplementary material:**

The online version of this article (10.1007/s10567-018-0253-x) contains supplementary material, which is available to authorized users.

## Background

It is estimated that anywhere between 13 and 32% of children and young people (0–19 years) suffer from a chronic or life-limiting condition (Fraser et al. 2012; Van Cleave et al. 2010; Wijlaars et al. 2016). A chronic childhood illness can be defined as one that occurs between the ages of 0 and 18 years, is medically diagnosed and reproducible using valid methods or instruments, has been present for longer than 3 months or has occurred three or more times in the past year and is likely to reoccur, and is not (yet) curable or is highly resistant to treatment (including mental health conditions) (Mokkink et al. 2008). This definition encompasses both physical and mental health disorders. The World Health Organisation defines a mental health disorder as “generally characterised by a combination of abnormal thoughts, perceptions, emotions, behaviour and relationships with others”. Mental Health disorders include depression, bipolar affective disorder, schizophrenia, dementia, intellectual disabilities and developmental disorders including autism (WHO 2017). Hence, for the purposes of this review, developmental disorders including autism are included within the category of mental health disorders. It is estimated that around 54.6%^1^ of families have two or more children (OECD 2016), which means that approximately 7–17% of children have a sibling with a chronic illness.

### Consequences

Previous studies that have investigated the impact of having a sibling with a chronic illness have produced inconsistent results. Some literature suggests an elevation in psychological distress and mental health disorders in siblings (Cadman et al. 1988), and other studies suggest such siblings do not warrant further investigation (Bischoff and Tingstrom 1991). Much of the research has adopted a disease-/disorder-specific approach; some have taken a “non-categorical” or “broad” (Stein et al. 1993; Stein and Jessop 1982) approach, in which they do not differentiate based on the chronic illness (Cadman et al. 1988). Having a sibling with a chronic illness in the family can result in an imbalance of resources, such as time spent with their parents. It can also pose social challenges (Bluebond-Langner 1996) and has been noted to have a negative impact on educational attainment (Breining 2014). Family dynamics can be stressed and can regularly result in tension between parents, along with a lack of socialising outside of the family (Kvist et al. 2013; Mailick Seltzer et al. 2001).

A meta-analysis of 51 studies looking at the psychological impact of having a sibling with a chronic illness found a significant overall negative impact and specifically a significant negative impact on psychological functioning, peer activities and cognitive development (Sharpe and Rossiter 2002). Sharpe and Rossiter’s (2002) meta-analysis only included studies which considered the siblings of children with a chronic physical health condition. Their findings are consistent with the findings in a more recent meta-analysis, which included 13 additional studies (Vermaes et al. 2012) and found a significant, although small, negative effect on psychological functioning. Prior to their 2002 meta-analysis Rossiter and Sharpe also published a meta-analysis which considered siblings of children with mental retardation (Rossiter and Sharpe 2001). Their findings indicated that these siblings also have a significantly lower psychological functioning.

Psychological function is defined as “an individual’s ability to achieve their goals, both within themselves and in the external environment. This includes their emotions, behaviour (both internalising and externalising behaviours), social skills and their overall mental health” (Preedy and Watson 2010). Behaviour is typically measured using tools such as the Childhood Behavioural Checklist (CBCL) (Achenbach 1991) or the Strengths and Difficulties Questionnaire (SDQ) (Goodman 1997). These self-reported tools measure internalising behaviours, e.g. emotional symptoms, and externalising behaviours, e.g. conduct problems, along with a total score of behavioural problems. Both the SDQ and the CBCL have been shown to be able to distinguish between psychiatric and non-psychiatric cases (Goodman 1997; Seligman et al. 2004). It has been noted that there is the potential for a greater impact on internalising behaviours in siblings. An increase in internalising behaviours, including anxiety and depression, have been observed both in studies that adopt a broad approach (e.g. Cadman et al. 1988) and those using a more disease-specific approach (Cadman et al. 1988; Fisman et al. 1996; Hastings 2003; Verté et al. 2003), and this is supported by previous meta-analyses (Rossiter and Sharpe 2001; Sharpe and Rossiter 2002; Vermaes et al. 2012). While evaluating a camp intervention for siblings of children and young people with a chronic illness, Sidhu et al. (2006) found that one-quarter of their sample suffered from psychological distress within the clinical range, and these were more internalising in nature. Suggestions as to why this may be include children not wishing to burden parents further (Sidhu et al. 2006), the quality of the family environment (Verté et al. 2003) and factors relating to the child themselves, e.g. age, sex (Hastings 2003).

Several studies have proposed a link between psychological functioning, e.g. anxiety, and a lack of understanding of a sibling’s chronic condition (Carpenter et al. 1990; Houtzager et al. 2001; Sidhu et al. 2006). It has been suggested that a limited understanding of their sibling’s illness can lead to poor adaption (Evans et al. 2001). It may also be that the lack of knowledge about their sibling’s illness negatively impacts the sibling relationship (Roeyers and Mycke 1995). Improving the child or young person’s understanding of the sibling’s condition has been linked to reduced anxiety levels (Houtzager et al. 2001). Strategies that adopt an educational approach therefore may help improve the mental health of siblings of children and young people with a chronic illness. In this way, knowledge of a sibling’s condition may be considered part of their psychological functioning.

Along with the potential negative psychological impacts noted, the literature exists that suggests positive effects of having a sibling with a long-term condition. For instance, within Rossiter and Sharpe’s original meta-analysis, sibling relationship was found to positively moderate the level of psychological distress in siblings of children with mental retardation (Rossiter and Sharpe 2001); however, in their subsequent meta-analysis this relationship was insignificant (Sharpe and Rossiter 2002). Other suggested positive impacts include an increase in maturity (Grossman 1972), warmth and understanding towards their sibling (Fisman et al. 1996), and prosocial behaviour (Ferrari 1984; Lobato et al. 1988). There has been little investigation into these positive findings, but it has been suggested that they may act as protective factors for mental health outcomes in children and young people (Fisman et al. 1996). Parents may be unaware of the positive interactions occurring between the siblings, perhaps due to the salience of negative interactions (Rivers and Stoneman 2003), and such lack of awareness could potentially influence parental reports which are often used in evaluations.

### Predictive Factors

Identifying siblings of children and young people with a chronic illness that are at greatest risk of poor psychological functioning could help to target interventions. Targeting interventions at those who are in greatest need is imperative to resource-limited services. Family-related factors have been suggested that may help identify those at risk. For instance, Daniels et al. (1987) found that less family cohesion and expressiveness were related to an increased psychological risk in siblings. Positive family functioning has also been noted as a potential protective factor in children with siblings with Down’s syndrome, yet not in those with pervasive developmental disorder (Fisman et al. 1996). It has also been found that siblings of children receiving treatment for mental health problems were more likely to live in poorly functioning families (Barnett and Hunter 2012). When considering potential predictive factors in their meta-analysis, Vermaes et al. (2012) reported that gender, birth order or diagnosis was not significantly associated with behavioural problems. They did, however, find that when the child has a chronic condition that is associated with a higher mortality rate and more intrusive treatment, the siblings were significantly more likely to have greater internalising and externalising problems, along with less positive self-attributes.

### Well-being Interventions

The definition of well-being is continually developing. It is suggested that high well-being is positively related to good mental health and can be made up of the following ten components: competence, emotional stability, engagement, meaning, optimism, positive emotion, positive relationships, resilience, self-esteem, and vitality (Huppert and So 2013). Well-being interventions have been suggested to help improve psychological outcomes (including anxiety, depression, stress, self-esteem and coping) of siblings of children and young people with a chronic illness. These interventions have taken various forms, including group interventions (Heiney et al. 1990; Houtzager et al. 2001; Lobato and Tlaker 1985; Smith and Perry 2005), sibling training (Ferraioli et al. 2012), camps (Kiernan et al. 2004; Sidhu et al. 2006) and family-based support (Besier et al. 2010; Giallo and Gavidia-Payne 2008). A range of populations have been targeted; some have been disease-specific (Dolgin et al. 1997), while others have taken a broad approach (Cadman et al. 1988). The content and duration of the interventions are highly varied. Camp interventions are typically formulated from the concept of therapeutic recreation (Fine and Fine 1996), which focuses on enjoyment and freedom in recreation, while other studies, particularly those involving a group interventions, have utilised more psychoeducational components (Giallo and Gavidia-Payne 2008; Granat et al. 2012; Lobato and Kao 2002).

### Evaluating Interventions

Evaluations of interventions are limited and typically associated with methodological issues including small sample sizes (Marszalek et al. 2011), a lack of intervention integrity tracking (Kryzak et al. 2015), and large heterogeneity (Ali et al. 2014). A previous systematic review that considered interventions for siblings of children with a chronic illness or disability included articles published between 1985 and 2008, adopted a broad approach and included 14 papers (Hartling et al. 2014), although the definition of chronic illness or disability in this review was unclear. Hartling et al.’s review found a large inconsistency in treatment effects on behavioural and emotional outcomes and highlighted the importance of the sensitivity of the measures used as several of the included studies reported the child to be within the “normal” range of mental health prior to the intervention. It is suggested that this may cause a ceiling effect on results as their scores are unlikely to continue to improve beyond their current point. A more recent review by Tudor and Lerner (2015) included 16 papers looking at interventions for psychological functioning targeted specifically at siblings of children with developmental disabilities (DD). Tudor and Lerner initially argued that the experience of typically developing siblings of children with DD was distinguishable from siblings of children or young people with physical disabilities, yet within their conclusion they acknowledged that the best services for siblings may not make that distinction. Therefore, due to a lack of clarity in the advantages of interventions for siblings of individuals with either a physical or psychological disorder, this systematic review and meta-analysis includes both populations.

In summary, previous systematic reviews have suggested that there is a need for interventions to improve psychological well-being in siblings of children and young people with a chronic illness, but limited evidence has been provided about the effectiveness of interventions that are offered to siblings of children with either a physical or mental health condition. When considering this subject it is important to remember that children with a physical health condition have an increased likelihood of having a mental health condition (Lavigne and Faier-Routman 1992) and that there is a close relationship between physical and mental health. There have been calls for physical and mental health to be more closely integrated (Prince et al. 2007; Scott et al. 2016). In general, there is an absence of evidence on the effectiveness of interventions for siblings and a lack of clarity regarding the requirement for interventions to distinguish between siblings of children with a physical or mental health condition. There is value in investigating the effectiveness of the interventions regarding the psychological well-being of siblings of children and young people with either a chronic physical or mental illness (or both). Additionally, no meta-analysis has been conducted on the effectiveness of psychological interventions on siblings of children and young people with chronic illness. The aims of this review are to:

1. Conduct a systematic review to synthesise the literature that evaluates well-being interventions offered to siblings of children and young people with a chronic illness.
2. Conduct a meta-analysis to quantitatively evaluate the impact of the evaluations included in the systematic review.

## Methods

### Sources and Search Strategy

A systematic search was conducted, following the Preferred Reporting Items for Systematic Reviews and Meta-Analyses (PRISMA) guidelines (Moher et al. 2009) and Cochrane recommendations (Higgins and Green 2011). Electronic database searches were completed along with reference list and citation hand searches, and grey literature searches. The following databases were used: PsycINFO, EMBASE, CINAHL, PubMed, Scopus and Web of Science, and PsycExtra was used to search for grey literature. The search strategy was piloted in November 2016, and following review was re-run in January of 2017, by three independent researchers (MMS, CR and LC), to include all literature up to the end of 2016. The search strategy was built using the Participant, Intervention, Comparator, Outcome (PICO) framework, as suggested in PRISMA guidelines (Shamseer et al. 2015). The broad themes included in the search strategy were sibling, chronic condition, intervention and mental health. The search strategy was adapted to each database. The full search strategy can be found in Online Resource 1.

### Study Selection

Studies were included if they evaluated an intervention offered to siblings of children and young people with a chronic health condition, as defined previously. It was required that the two children live together (or were of an age where it is assumed they would still live together, i.e. below 18 years of age). The sibling must be considered “healthy” themselves and not a donor for the ill child or young person.

The intervention could take any form, provided it aimed to improve the psychological well-being of the sibling, and reported an outcome that is related to the mental health of the sibling including direct psychological outcomes, e.g. anxiety, depression and stress, as well as related factors, e.g. knowledge, social support, self-esteem, relationships, coping and adjustment. Family-level interventions were not included unless there were sufficient (at least one) sibling-specific outcomes, as described above, reported.

Included studies could be mixed methods if they report the result of at least one quantitative measure. Any form of trial was accepted if it evaluated the effectiveness of the intervention; this included pre-post design trials.

Studies were excluded if unavailable in English or French. Studies that involved bereaved siblings and studies that looked specifically at sibling donors were also excluded.

### Risk of Bias Assessment

The Effective Public Health Practice Project Quality Assessment Tool (EPHPP) (Thomas et al. 2004) was used to evaluate the quality of all papers included in this review. This tool was chosen as it has been shown to have a higher inter-rater reliability relative to the Chronic Collaboration Risk of Bias Tool (Armijo‐Olivo et al. 2012) and is appropriate for use across different study designs compared to other tools such as the ROBINS-I, which is only appropriate for non-randomised trials (Sterne et al. 2016). This allowed confidence in the consistency and reliability of the assessments.

The EPHPP evaluates studies on eight components: selection bias, study design, confounders, blinding, data collection methods, withdrawals and dropouts, intervention integrity and analyses. The ratings for all but intervention integrity and analyses are combined to give the study an overall rating of strong, moderate or weak.

Searches, study selection, quality assessment and data extraction were completed by three independent researchers: MMS, CR and LC. Any discrepancies were dealt with through discussion, and if a consensus could not be reached, the opinion of an additional independent researcher was sought.

### Data Extraction and Analysis

A data extraction form was created, using the Effective Practice and Organisation of Care (EPOC) data collection form (EPOC 2013) as a base, to ensure sufficient data were collected from each study included in the systematic review and meta-analysis.

It was expected that there would be large heterogeneity in outcome measure used across studies; therefore, a random-effects meta-analysis was conducted. A standardised mean difference (SMD) and restricted maximum likelihood (REML) technique was used to estimate effect sizes and weights in STATA 14 (StataCorp 2015). The SMD was estimated using Hedge’s g technique, allowing for a smaller sample size relative to Cohen’s d method, which is typically used in meta-analyses in this subject area (Cuijpers 2016).

The SMD technique allows the combination of different scales that are measuring the same outcome. For instance, the Strengths and Difficulties Questionnaire (SDQ) (Goodman 1997) and the Child Behaviour Checklist (CBCL) (Achenbach 1991) both measure behavioural outcomes and have been noted to have highly correlated scores (Goodman and Scott 1999).

This review was registered on PROSPERO (International Prospective Register of Systematic Reviews), registration number CRD42017056740.

## Results

### Search Results

**Fig. 1:**
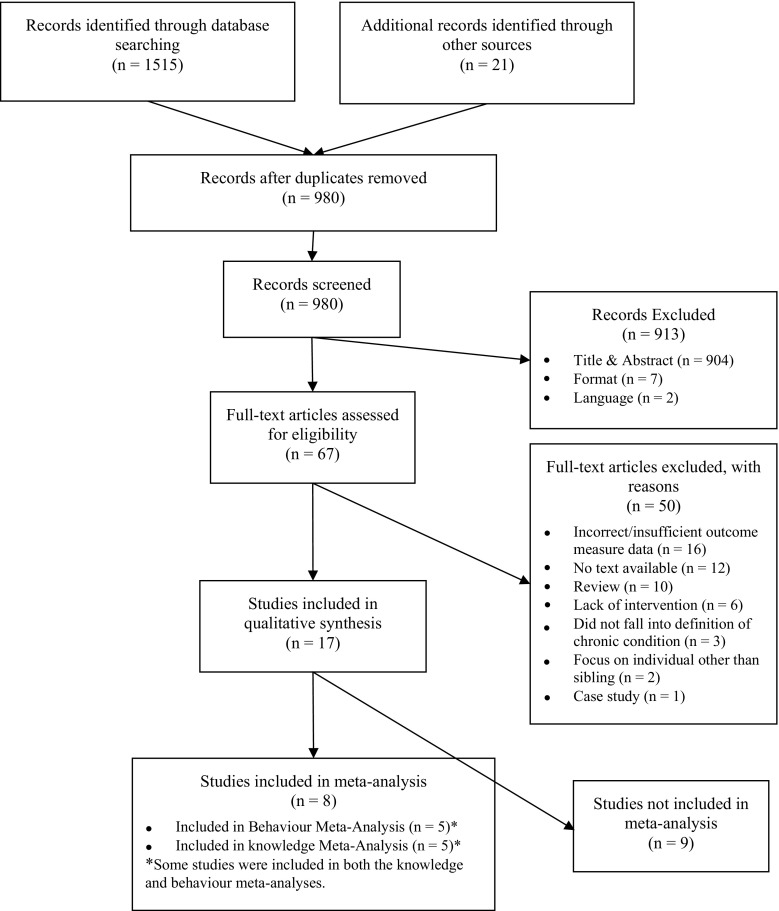
PRISMA flow diagram

A total of 1536 papers were identified from the initial searches. After removal of duplicates 980 records were screened. Of that, 913 were excluded based on title and abstract (*n* = 904), format (*n* = 7) and language (*n* = 2). Sixty-seven full-text articles were assessed for eligibility, and 17 were included in the qualitative synthesis. Eight studies were included in the meta-analysis. Five of the papers included reported an outcome measure of behaviour, and five reported on knowledge change following intervention. The nine papers not included in the meta-analysis either did not report sufficient data, used a different study design, or did not use either a behaviour or knowledge outcome measure. The flow of papers through the process of eligibility can be seen in Fig. 1. In the initial search, ten reviews were identified including three systematic reviews (Hartling et al. 2014; Prchal and Landolt 2009; Tudor and Lerner 2015). Rather than including the reviews, as there were discrepancies with the inclusion criteria, it was decided that the individual papers from each should be reviewed against the eligibility criteria.

### Baseline Characteristics

Across the 17 included studies, there were 1264 participants. Age of participants ranged from 6 to 15 years, with an average of 10.47 years.^2^ There was a relatively even gender balance in the overall sample, with 53% of participants being female. Further demographic information can be found in Table 1.

**Table 1:** Summary of demographics

Author(s)	Year	Total n	Experimental n	Control n	Other n (specified)	Age, years mean (SD) or range	Gender (% Female)	Siblings condition
*Physical health conditions*								
Besier et al.	2010	259	259	N/A	N/A	8.6 years (3.3)	45.6%	Cystic fibrosis (20.5%)
								Congenital heart disease (31.7%)
								Cancer (49%)
Dolgin et al.	1997	23	23	N/A	N/A	11.7 years (3)	48%	Cancer (100%)
Heiney et al.	1990	14	7	7	N/A	9–15 years	57.1%	Cancer (100%)
Houtzager et al.	2001	24	24	N/A	N/A	11.3 years (3.1)	63%	Cancer (100%)
Kiernan et al.	2004	119	119	N/A	N/A	11.5 years (2.4)	45%	Cancer (52.2%)
								Haematological-related illness (21.7%)
								Not reported (26.1%)
Sidhu et al.	2006	26	26	N/A	N/A	8–13 years	52%	Acute lymphoblastic leukaemia (65%)
								Acute myeloid leukaemia (7.6%)
								Brain tumours (7.6%)
								Neuroblastomas (7.6%)
								Osteogenic sarcoma (3.8%)
								Hepatoblastoma (3.8%)
								Ependymoma (3.8%)
*Mental health conditions*								
Evans et al.	2001	28	28	N/A	N/A	6–12 years	68%	Learning disabilities and associated challenging behaviour
Kryzak et al.	2015	15	15	N/A	N/A	6–14 years	40%	ASD (100%)
Phillips	1999	180	90	90	N/A	9–12 years	60%	Mild mental retardation Moderate mental retardation
Smith and Perry	2005	26	26	N/A	N/A	10.6 years (2.1)	54%	Autism
*Combined*								
Cebula	2012	132	45	45	26 (post-ABA and post-ABA control)	ABA: 9.1 years (2.4)	ABA: 53%	Autism: ABA (80%); ABA control (73%); post-ABA (88%); post-ABA control (85%)
						ABA Control: 9.3 years (3.3)	ABA control: 60%	Other (Asperger’s, ASD, HFA): ABA (20%); ABA Control (27%); post-ABA (12%); post-ABA control (15%)
						Post-ABA: 9.9 years (2.3)	Post-ABA: 42%	Additional diagnoses: ABA (16%); ABA control (13%); post-ABA (15%); post-ABA control (15%)
						Post-ABA control: 9.7 years (3.1)	Post-ABA control: 46%	
D’Arcy et al.	2005	16	16	N/A	N/A	8–10 years	45.5%	Disability (a physical or intellectual; disability, or a combination of both)
Giallo and Gavidia-Payne	2008	21	12	9	N/A	Intervention: 11.75 years (2.9)	57.1%	Down syndrome (19%)
								Autism (23.8%)
								ADHD (4.8%)
								Polymicrogyria (4.8%)
								Multiple disabilities (14.3%)
						Control: 11.00 years (2.3)		Cystic fibrosis (4.8%)
								Congenital heart disorder (9.5%)
								Multiple illnesses (4.8%)
								Williams syndrome (9.5%)
Granat et al.	2012	54	54	N/A	N/A	8–12 years	61%	ADHD (16.7%)
								Asperger syndrome (13%)
								Physical disability (14.8%)
								Intellectual disability (31.5%)
								Autism (24.1%)
Lobato and Kao	2002	54	54	N/A	N/A	8–13 years	56%	Physical disabilities (26%)
								ASD (23%)
								Mental retardation (21%)
								Medical disorders (17%)
								Combined psychiatric and learning disorders (13%)
McLinden et al.	1991	11	6	5	N/A	9.8 years (2.2)	64%	Mentally retarded (45%)
								Physically handicapped (9%)
								Multiply handicapped (45%)
Williams	2003	252	79	102	Partial treatment: 71	Intervention: 11.1 years (2.2)	50%	Cystic fibrosis (4.4%)
								Diabetes (34.9%)
						Partial: 11 years (2.5)		Spina Bifida (9.5%)
								Cancer (8.7%)
						Control: 11.2 years (2.5)		Developmental disabilities (42.5%)

### Quality Assessment

Of the included studies, eight (47%) were considered of weak quality (Besier et al. 2010; D’Arcy et al. 2005; Evans et al. 2001; Giallo and Gavidia-Payne 2008; Heiney et al. 1990; Houtzager et al. 2001; Kiernan et al. 2004; McLinden et al. 1991), seven (41%) were rated as moderate (Cebula 2012; Dolgin et al. 1997; Granat et al. 2012; Kryzak et al. 2015; Lobato and Kao 2002; Phillips 1999; Williams et al. 2003) and only two (12%) were rated as strong (Sidhu et al. 2006; Smith and Perry 2005). The two strong studies were both rated strong in the confounders, data collection methods, and withdrawals and dropouts components of the EPHPP, and moderate in the remaining three components. The one RCT study included in the review was rated weak overall (Giallo and Gavidia-Payne 2008). One paper was scored N/A for withdrawals and dropouts as it had one time point only, and therefore, quality assessment in this area was irrelevant for this paper (Cebula 2012). A table of quality assessment results can be found in Online Resource 2.

### Interventions

Nine of the 17 studies included in this review were group-based interventions (53%); the next most frequent form of intervention was camp-based interventions (18%). The studies were conducted in mainly high-income, predominately Caucasian countries including Germany, UK, The Republic of Ireland, Australia, Canada, the Netherlands and Sweden. The largest number of studies coming from one location was four, which were all based in the USA. The duration of the interventions ran from 4 days (Sidhu et al. 2006) to 96 months (Cebula 2012), with a median and mode duration of 6 days. There was little consensus in the approach taken in the interventions, even between studies that used similar designs. Further details about the included interventions can be found in Table 2.

**Table 2:** Intervention details

Author(s)	Year	Name of Intervention	Who delivered intervention	How often/how many sessions were involved	Sessions	Protocol available	Protocol adherence recorded	Intervention offered to other members of family
*Physical health conditions*								
Besier et al.	2010	Family-oriented rehabilitation programme	Psychosocial team	4-week programme. Session offered 1–3 times per week	Psychoeducational group, exercise, relaxation, supportive/psychotherapy, parent–child sessions	Individually arranged treatment protocols	N/A	Ill child admitted for rehabilitation. Parents also treated according to individually arranged protocols
Dolgin et al.	1997	Structured group intervention	Clinical social worker, a child life specialist and a supervising psychologist	Six group sessions were held on consecutive weeks	In addition to group discussions concerning their experience of the illness and its impact, subjects took part in arts and crafts and other creative activities in order to encourage interaction among participants and to promote non-verbal expression of relevant feelings and themes	Detailed structure available	Unclear	No
Heiney et al.	1990	Sibling support group	Co-therapists: a fellow in child psychiatry, and a pediatric oncology nurse	Seven 1-h sessions	The group was organised so that each session focused on a specific topic: introduction and orientation, diagnosis, treatment, school, coping, family relationships, and the future	No	No	Concurrent parent group
Houtzager et al.	2001	Support group for siblings	Led by two well-trained psychologists	Five weekly sessions	First session: getting to know each other, second session: changes, third session: emotions related to illness, fourth session: paediatric oncologist invited to talk, final session: siblings visit the oncology ward	No	No	No
Kiernan et al.	2004	The Barretstown gang camp	Unclear/camp Staff	10-day sessions	Core activities: music, theatre, photography, arts and crafts, wordsmith, woodwork, canoeing, fishing, horse-riding, adventure, archery and camping. Periphery Activities: hangout, and evening activities. Social Activities: Cottage chat, rest hour and the opportunity to meet other from different countries (1)	No (more info: https://www.barretstown.org/)	No	Camp for children with life threatening illnesses and their siblings
Sidhu et al.	2006	Camp onwards	Group facilitators (undertook pre-camp training workshop)	4-day	The program aimed to provide an opportunity to develop peer support networks and social competencies; provide age appropriate information on cancer, treatment and its impact on all the family; facilitate activities, that encourage the expression of feelings; and impart strategies to enhance adjustment to the family stressors in a safe environment	Manual (soon to be published at point of paper publication)	No	No
*Mental health conditions*								
Evans et al.	2001	Sibling support groups	Facing the challenge’ multi-disciplinary team. Comprised of nurses, a psychologist and outreach workers	Three consecutive full days, and then on a weekly basis for six evenings. A final day at a local theme park	Had a problem-solving focus. The majority of activities were of an educational and informative nature. Leisure activities were also used	No	No	No
Kryzak et al.	2015	The support and skills program (SSP)	Special education teacher, school councillor, volunteers, a psychology doctoral student	Seven 2 h sessions	Focused on developing a network of peers who face similar family challenges, learning about Autism Spectrum Disorders (ASD) and coping strategies	No	No	Support for Child with ASD
Phillips	1999	After-school program	Six team leaders (community centre staff), and seven volunteers	15-week, after-school (3–5:30 pm) every weekday	Group discussions, recreation and homework assistance	No	No	No
Smith and Perry	2005	Sibling support groups	Treatment, Research, and Education for Autism and Developmental Disorders (TRE-ADD) staff	Weekly for 8 consecutive weeks	Exercises, games, and activities that were fun and promoted group cohesion, providing information sessions on autism and related disorders, and facilitating discussion relating to feelings and attitudes associated with living with a brother or sister who has a developmental disability	No	No	No
*Combined*								
Cebula	2012	Applied Behavior Analysis (ABA)	Mother/partner, outside agency, or parents and outside agency	2-96 months/5–40 h per week	N/A	No	No	Intensive home-based intervention for child with autism.
D’Arcy et al.	2005	SibShops	Unclear. Clinical Psychologist conducted interviews	Once a month for four consecutive months	Consists of high and low energy activities, interspersed with discussion about disability and each sibling’s experiences	No—based on the model developed by Meyer and Vadasy (1994)	Unclear	No
Giallo and Gavidia-Payne	2008	Sibstars	A clinician with postgraduate psychology training	Weekly telephone support offered to sibling and parent for 6 weeks	After the first face-to-face session, each week families were required to read an information booklet and complete the practice activities provided	Yes	Programme adherence checklist was used	Involved Parents
Granat et al.	2012	Sibling group intervention	Clinical staff from an outpatient rehabilitation centre	A 2-h session every week for 6 weeks	Content intended to increase knowledge and problem-solving skills	A manual (in Swedish) for clinical practice was compiled	No	Separate education groups being provided for parents
Lobato and Kao	2002	Sib link	Doctoral level trainees in psychology or psychiatry	Six 90-min sessions	Activities alternated between explicitly focused “main events” and other more social-recreational activities	Manuals available on request	No	Parent group
McLinden et al.	1991	Sibling support group	School psychologists	Six weeks, 1-h per week	Focused on developing participants’ acceptance of both negative and positive feelings about their siblings. Information was provided and numerous activities were utilized.	No—based on Lobato (1985)	No	No
Williams	2003	Intervention for Siblings: EXPERIENCE Enhancement (ISEE)	Pediatric nurse clinicians	5-days	Structured teaching about the brother/sister’s illness, psychosocial session, a 5-day residential summer camp, and two booster sibling session and parent sessions	Brief Protocol Available	No	Parent sessions

**Fig. 2:**
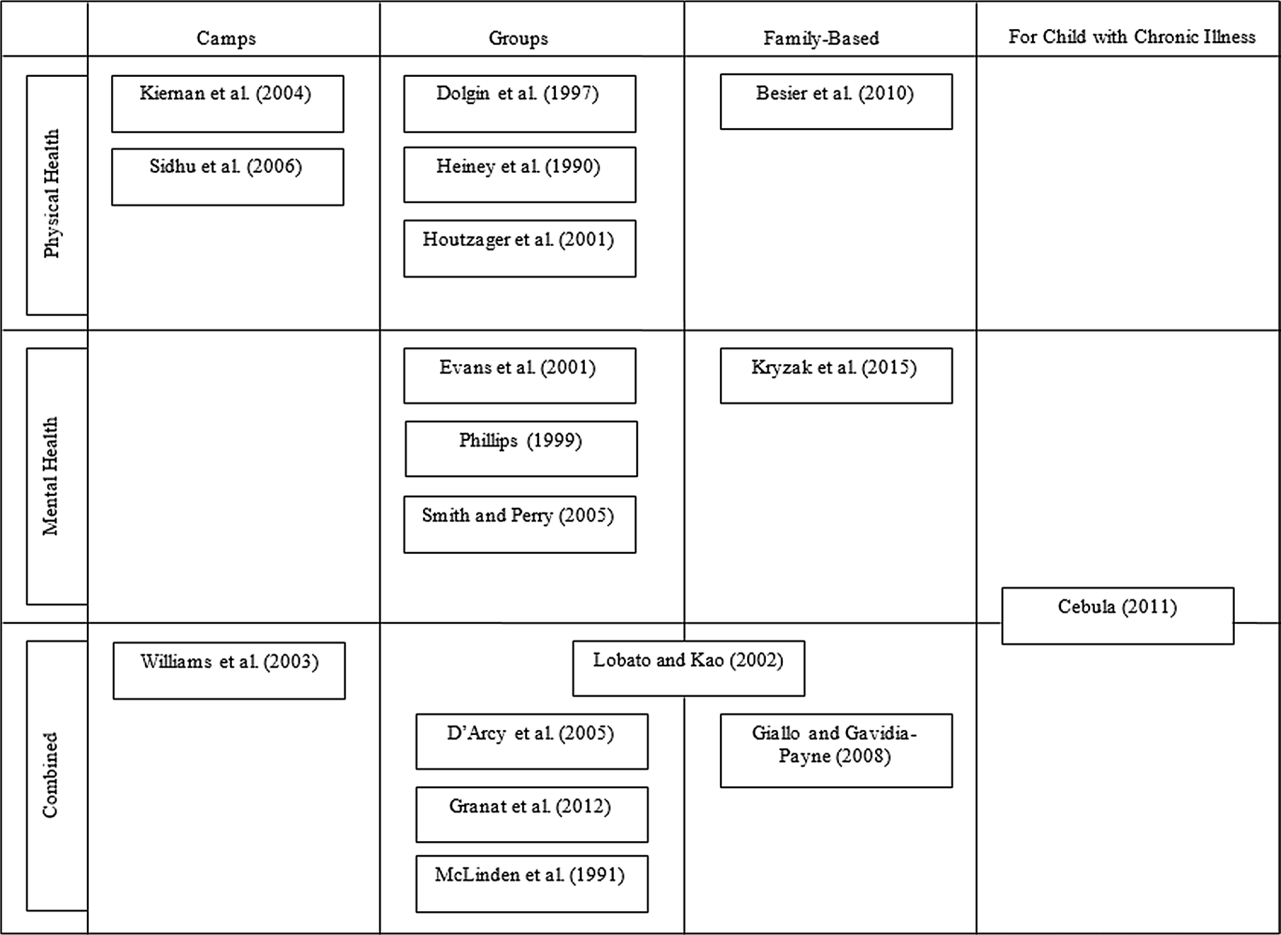
Types of intervention offered by condition group

Of the 17 papers included, six focused on physical illnesses, four focused on mental health conditions, and the remaining seven focused on a combination of physical and mental health conditions. Several studies focused on specific conditions; for instance, four of the 17 studies offered interventions to siblings of children with cancer. Of the six papers that focused on physical illnesses, much of their samples were made up of siblings of children with cancer (minimum 47.9%). A breakdown of the type of intervention offered by physical, mental or combined studies is shown in Fig. 2.

### Systematic Review

#### Physical Conditions

Of the six papers that focused entirely on chronic physical illnesses, four of them focused exclusively on cancer diagnoses, while the remaining two incorporated congenital heart disease, cystic fibrosis and other haematological-related illnesses. Across these six studies, there were nine different outcomes considered and 13 different measures used.

There was significant improvement in self-esteem (Kiernan et al. 2004; Sidhu et al. 2006), behaviour (Besier et al. 2010), knowledge, attitude and feeling, mood (Dolgin et al. 1997), and anxiety (Houtzager et al. 2001; Sidhu et al. 2006). Three studies examined quality of life (QoL), and all found significant improvements (Besier et al. 2010; Kiernan et al. 2004; Sidhu et al. 2006). There was no significant change in “coping” (Heiney et al. 1990) and affect (Kiernan et al. 2004).

#### Mental Health Conditions

Four of the 17 papers focused solely on children with a sibling with a chronic mental health condition. Two focused on autism spectrum disorders (Kryzak et al. 2015; Smith and Perry 2005), one on learning disabilities (Evans et al. 2001) and one on mental retardation (Phillips 1999). Across these four papers, ten outcomes were considered, and 12 different measures used.

There were significant improvements in self-esteem (Evans et al. 2001), sibling involvement (Evans et al. 2001), social support (Phillips 1999), anxiety and depression (Kryzak et al. 2015; Phillips 1999). There was no significant improvement in sibling interaction (Kryzak et al. 2015),^3^ sibling relationship, family functioning (Phillips 1999) or coping and adjustment (Smith and Perry 2005). Mixed results were noted from knowledge tests: Kryzak et al. (2015) found no significant improvement in Autism Sibling Knowledge, while a significant improvement in Autism knowledge was noted by Smith and Perry (2005). Evans et al. (2001) reported an improvement in knowledge about their siblings learning disorder but provided no statistical evidence.

#### Both Physical and Mental Health Conditions

Seven of the 17 papers did not specifically look at either physical or mental chronic illnesses. The study by Cebula (2012) evaluated the effectiveness of an intervention for siblings with autism, but noted that between 13 and 16% of these siblings had additional diagnoses of physical health conditions; therefore, their study is included in this section, rather than in the mental health focused section. Prevalence studies suggest that children with a chronic physical illness are more likely to have emotional/behavioural problems and psychiatric diagnoses (Hysing et al. 2007). The same is true of young people with autism where co morbidity is regularly found, including psychological difficulties and physical conditions (Matson and Goldin 2013). None of the included studies that examined only a mental or physical chronic health condition reported comorbidity, and they were therefore unable to consider how comorbidities may influence siblings psychological functioning.

Across these seven studies there were 15 outcomes considered, and 26 different measures used. Positive significant findings were found in the three studies that used a measure of “intervention impact” (Cebula 2012; McLinden et al. 1991; Williams et al. 2003), in the two that evaluated coping and adjustment (Giallo and Gavidia-Payne 2008; Lobato and Kao 2002), and in the studies that looked at stress, family functioning (Giallo and Gavidia-Payne 2008), mood (Williams et al. 2003) and connectedness (Lobato and Kao 2002). Non-significant findings were found in the two studies that considered parent-related variables (Cebula 2012; Giallo and Gavidia-Payne 2008). Of the seven papers, five considered behaviour (Cebula 2012; Giallo and Gavidia-Payne 2008; Lobato and Kao 2002; McLinden et al. 1991; Williams et al. 2003), four evaluated the impact on self-esteem (Cebula 2012; D’Arcy et al. 2005; McLinden et al. 1991; Williams et al. 2003) and knowledge (Granat et al. 2012; Lobato and Kao 2002; McLinden et al. 1991; Williams et al. 2003), while two looked at attitude and feelings (McLinden et al. 1991; Williams et al. 2003) and sibling relationship (Cebula 2012; McLinden et al. 1991), and one considered social support (Cebula 2012), all of which produced mixed evidence.

#### Comparison

Coping and adjustment, knowledge and self-esteem were the only outcomes considered in all three categories (mental health, physical health and combined). Both Giallo and Gavidia-Payne (2008) and Lobato and Kao (2002) looked at a combination of health conditions and found a significant improvement in coping and adjustment, whereas Smith and Perry (2005) and Heiney et al. (1990), who considered mental health and physical health, respectively, found no significant improvement. Both of the studies that found significant improvements in coping and adjustment involved the parents of the sibling, which may indicate that, although both studies were combined studies, this finding may be explained by factors other than the consideration of combined physical and mental health conditions. The results for knowledge were spread across the types of study, and there appeared to be no clear divide across physical, mental health or combined studies. Self-esteem was considered in nine papers, of which six found significant improvements following intervention (Evans et al. 2001; Kiernan et al. 2004; Phillips 1999; Sidhu et al. 2006; Smith and Perry 2005; Williams et al. 2003), and three did not (Cebula 2012; D’Arcy et al. 2005; McLinden et al. 1991). The three that found no significant associations were all combined studies, and only one of the six significant results was a combined study (Williams et al. 2003). It is unclear whether this is due to study design. In the six papers that found a significant association, three were camps (Kiernan et al. 2004; Sidhu et al. 2006; Williams et al. 2003), and three were group support (Evans et al. 2001; Phillips 1999; Smith and Perry 2005). Two of the papers that did not find significant associations were also a group support evaluation (D’Arcy et al. 2005; McLinden et al. 1991), and one was a service for the ill child or young person (Cebula 2012).

#### Efficacy and Effectiveness

Due to the large heterogeneity in outcomes, small sample sizes and, in some instances, poor study design, it is challenging to compare the efficacy and effectiveness of the interventions. The only RCT included in this review (Giallo and Gavidia-Payne 2008) had a sample size of only 21 across both treatment (12) and control (9). They offered a family-based psychoeducational-based intervention called SibStars. Using seven outcome measures (including an evaluation of the intervention), they found a significant improvement in stress, coping and adjustment, the emotional symptoms subscale of the SDQ (behaviour) and family functioning.

As 11 of the 17 studies (65%) adopted a within-subjects pre-post design, without the use of a control, the results of these papers should be carefully interpreted and no assumptions of causality can be made. Within the discussion of two of the studies, consideration was given to the value of time spent together between the child and parent as a by-product of the intervention, but this was not accounted for this in their analysis (Houtzager et al. 2001; Williams et al. 2003).

#### Meta-Analysis

There were eight papers that could be included in the meta-analysis since they reported on the same outcomes using the same study groups and time points (Sutton et al. 2000). Of these eight studies, three were group interventions, four were family-based interventions, and one was an intervention for the child with the health condition. Two studies looked at mental health, two looked at physical health, and four looked at a combination of the two. These numbers were too small to conduct a subgroup analysis in this meta-analysis, so the studies were only considered for their results relating to behaviour (internalising, externalising and total score) and knowledge.

#### Behaviour

**Fig. 3:**
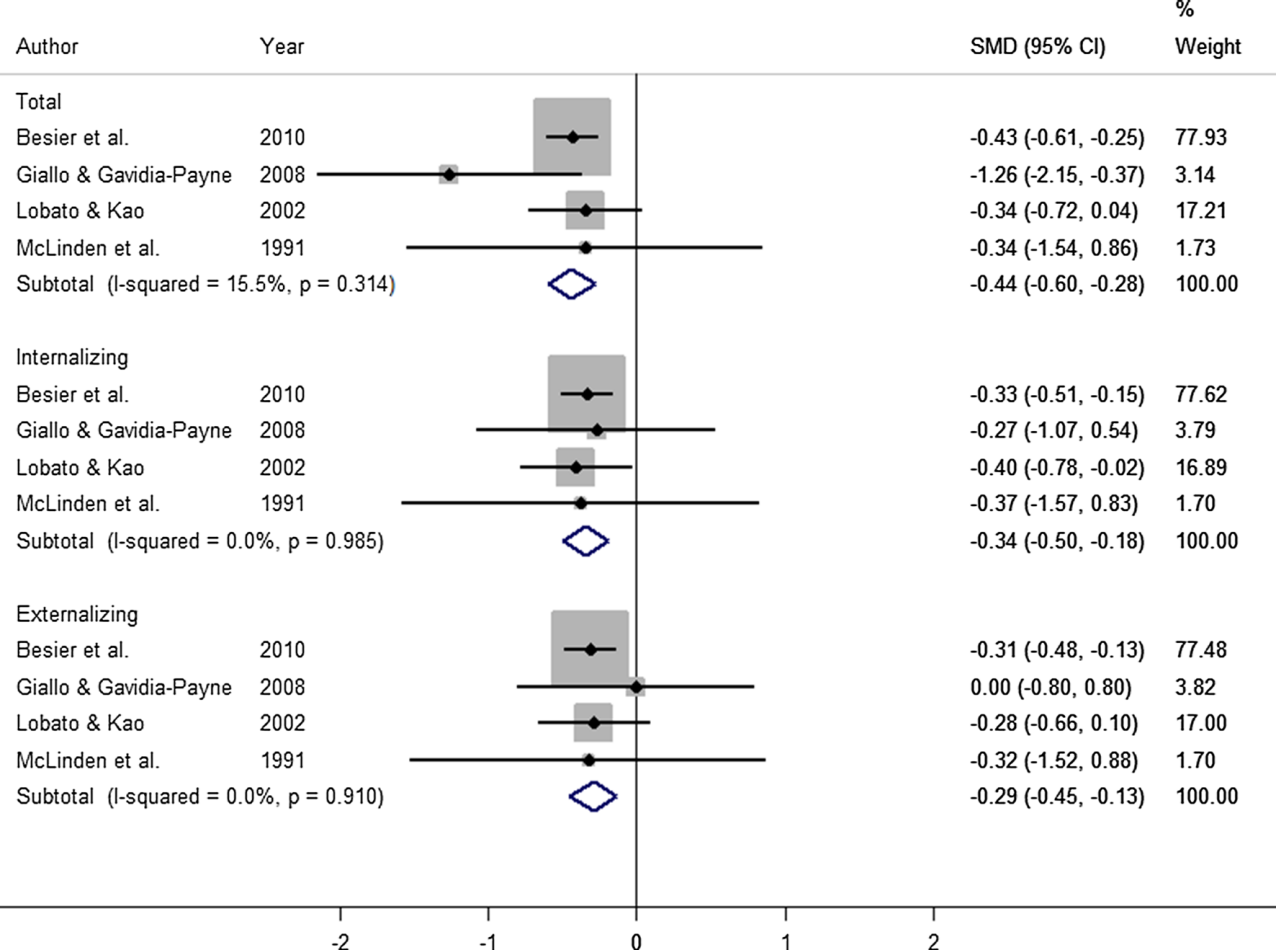
Forest plot of pre-post behaviour results

**Fig. 4:**
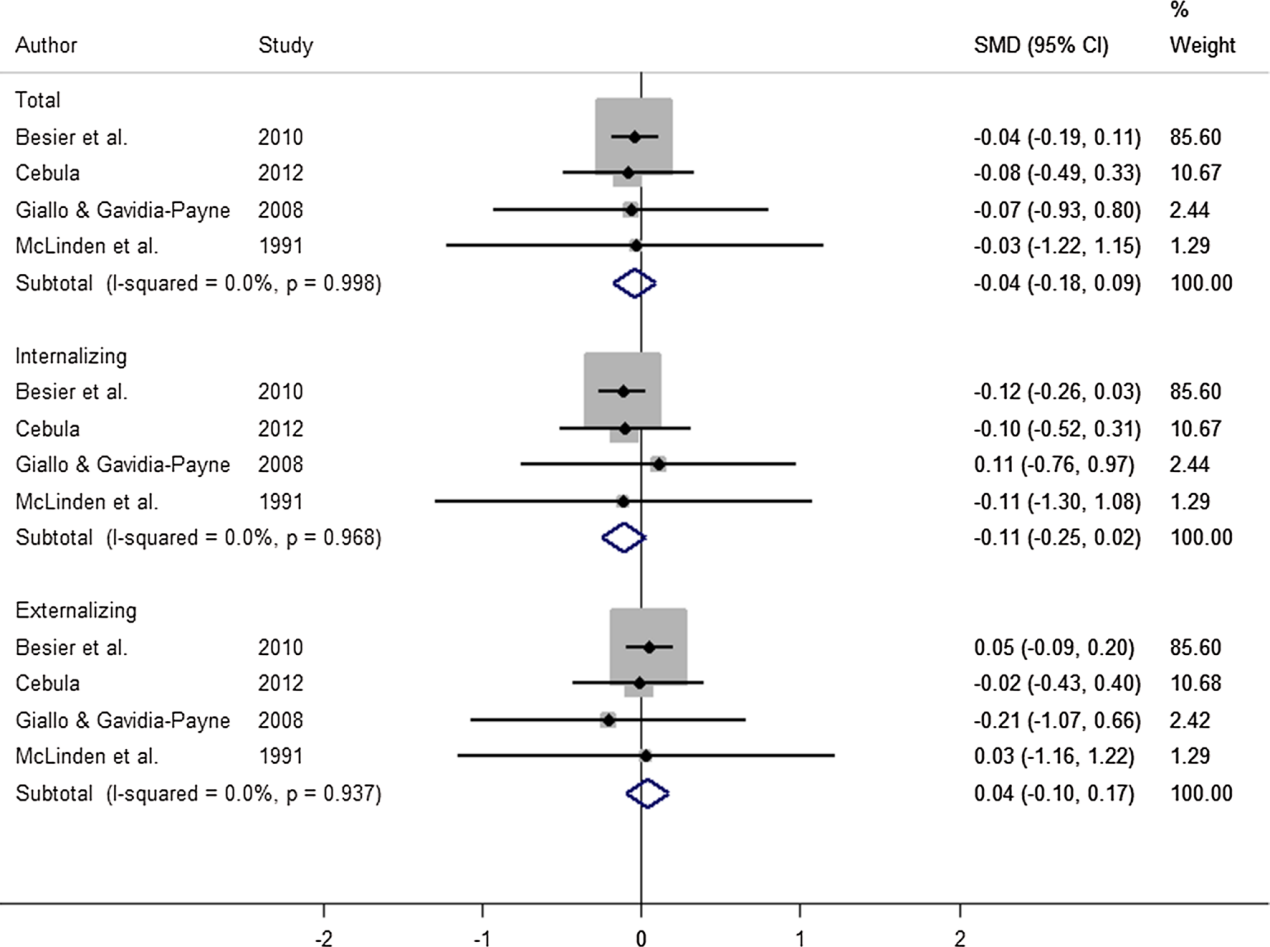
Forest plot of treatment (Tx)–control (Cntrl) behaviour results

The results of the SMD meta-analysis on behaviour was split into three categories: internalising, externalising and total score. Further, these papers were separated by whether they looked at pre-post measures in the treatment group or compared the intervention group post-treatment (Tx) to a control (Cntrl) group. Five papers were included in the analysis. The forest plots for each of the analyses can be found in Figs. 3 and 4. Cebula (2012) reported the SDQ from three different subjects (child, parent and teacher), to be consistent with other studies; only the parent report was included in the meta-analysis.

The pooled SMD estimates in the pre-post analysis (Fig. 3) all indicated improvement in behavioural outcomes on both SDQ and CBCL (as reflected through a reduction in score, relative to the baseline score). The pooled SMDs were as follows: internalising (SMD = − 0.34 [95% CI (− 0.50, − 0.18); *p* < 0.001]), externalising (SMD = − 0.29[95% CI (− 0.45, − 0.13); *p* < 0.001]) and total score (SMD = − 0.44 [95% CI (− 0.6,− 0.28); *p* < 0.001]). In contrast, the meta-analysis of studies comparing a treatment group to a control group resulted in no significant difference in any of the scales of behavioural difficulties considered (Fig. 4).

#### Knowledge

**Fig. 5:**
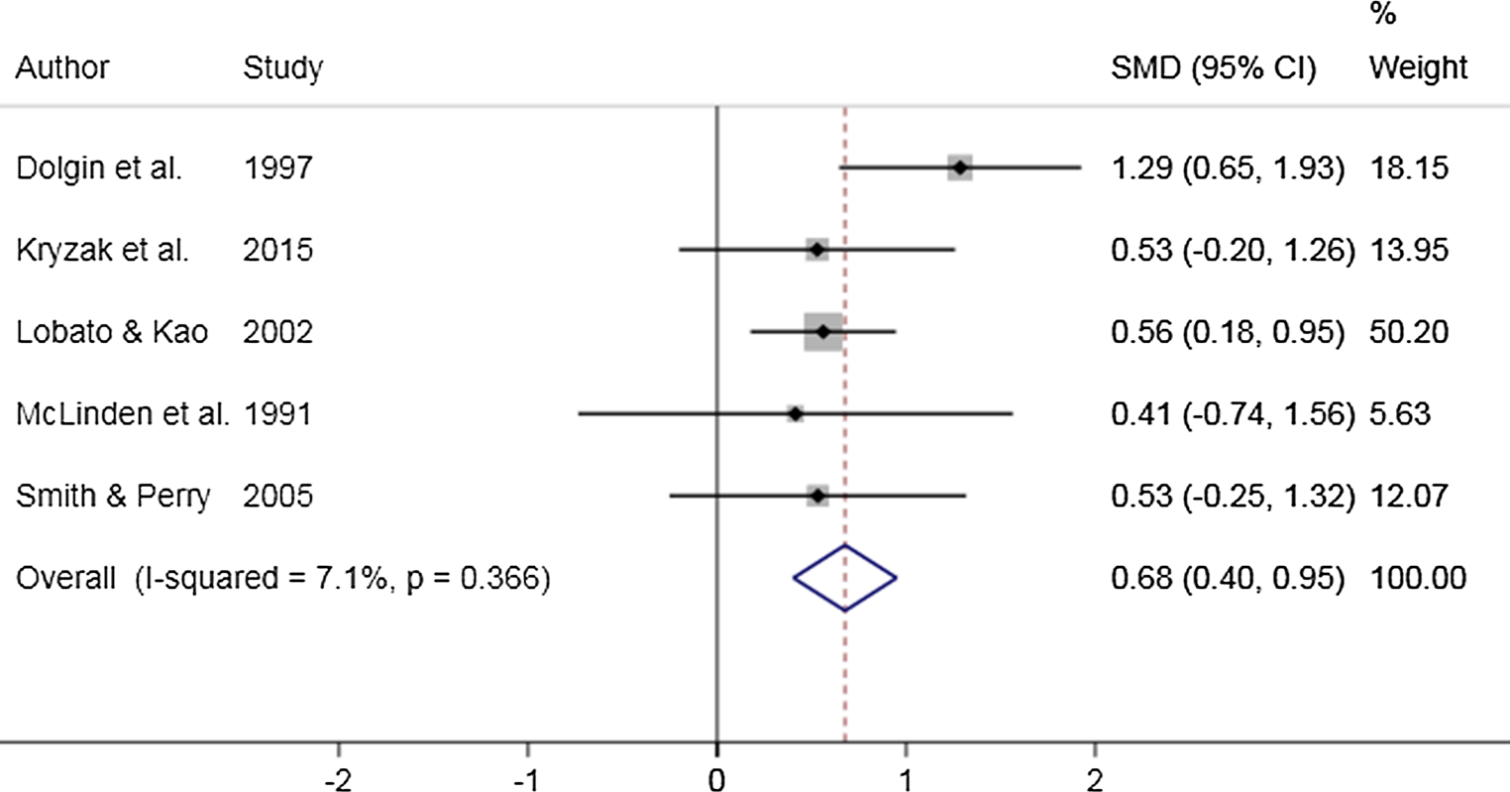
Forest plot of meta-analysis of knowledge outcomes

No two papers used the same measure for knowledge. The results of the meta-analysis that included five studies demonstrated that overall there was a small significant improvement in knowledge following the intervention. Only one paper (McLinden et al. 1991) reported control group results; therefore, this study design was not considered in this meta-analysis; rather, all studies included used a pre-post treatment study design. The results of this analysis can be seen in the forest plot shown in Fig. 5. The pooled SMD estimate for knowledge improvement following intervention is 0.68 [95% CI (0.40, 0.95); *p* < 0.001].

#### Bias

Bias in a meta-analysis may be attributed to a range of sources including reporting biases, poor methodological design or chance. Typically, a funnel plot would be used to test for the presence of bias. A funnel plot plots the treatment effect (SMD) against study precision [standard error (se)]. If the funnel plot is not symmetrical within the 95% confidence interval, it is taken as a sign that there is bias present. To ensure that the asymmetry is not attributable to chance, it is recommended to conduct a test for funnel asymmetry, such as Egger’s Test (Egger et al. 1997). However, this is not recommended if there are fewer than ten studies included in the analysis, as this is unlikely to distinguish true bias from chance due to low test power (Sterne et al. 2011). Therefore, due to the small number of studies included in this meta-analysis, no formal test for bias was completed.

## Discussion

The literature evaluating interventions for siblings of children and young people with a chronic illness is diverse and produces varied results. Studies included in this review involved siblings of children with a range of chronic health conditions, used various techniques to help improve the child’s psychological well-being and a range of measures to evaluate several outcomes. Each of these sources of heterogeneity provides significant challenges for conducting a systematic review and meta-analysis. The heterogeneity of interventions and samples makes it particularly challenging to make firm conclusions on the effectiveness of interventions using a pre-post study design. Despite the challenges, the current meta-analysis provided some evidence of effectiveness of interventions for siblings of children and young people with a chronic illness. The analysis of knowledge scores using pre-post measures indicated a small significant positive effect on knowledge following the intervention. Offering a knowledge component in an intervention could help facilitate the child or young person’s understanding of their sibling’s condition. By increasing understanding, it is possible the sibling will feel they have more control and this may help increase their coping skills (Heiney et al. 1990) although there has been relatively little work in this area.

When the treatment group was considered pre-post treatment, there was a significant improvement in their internalising and externalising behaviours, as well as total score (reflected by a reduction in scores). Yet when we consider treatment group post-intervention relative to a control group, there was no difference in behaviour scores. One suggestion as to why this may be is that the research process increased the salience of the needs of the sibling to parents in the control group which means their outcomes also improved. Data collected in the included studies did not allow for this factor to be considered. Future research should attempt to account for such contamination effects. Further explanations include a lack of difference from control at pre-treatment, low sensitivity of measures, or potentially a bias from parent-reported measures, consideration for each potential explanation will follow.

The sensitivity of the measures used in evaluations of this type should be further considered. For instance, a few of the included studies noted that the children are within the “normal” range before receiving the intervention, and this has the potential to cause a ceiling effect on gains from the intervention (Giallo and Gavidia-Payne 2008; Kiernan et al. 2004; McLinden et al. 1991). As previous meta-analyses (Rossiter and Sharpe 2001; Sharpe and Rossiter 2002; Vermaes et al. 2012) have found a significant negative effect on sibling’s psychological functioning, there appears to be a discrepancy between these samples and some of the samples considered in this review. This may be attributed to the samples themselves being unrepresentative or may be related to the measures used in these evaluations.

Another explanation as to why results differed by study design may be that the range in parent or sibling report measures resulted in either a downward (under-reporting) or upward (over-reporting) bias on the results. This single-rater bias has been noted in the literature (Rivers and Stoneman 2003). Within the papers included in this review, three consider the effect of parental reporting bias on results. Cebula (2012) compared the results given to the SDQ by parents, teachers and siblings and found that siblings view themselves significantly more negatively compared to their parents. Sidhu et al. (2006) recognised that parents are only able to report on the externalising behaviours of the sibling, whereas the sibling could report on their internalised problems, and their perceptions of these distresses were generally greater than parents. Finally, Lobato and Kao (2002) found a difference in ratings on the Sibling Perception Questionnaire (SPQ) and bring into question the sensitivity of parent-reported results using SPQ.

The positive impact of the interventions on behaviour may operate via a change in the parent–sibling dyad. For instance, parents may spend more time with siblings as a direct result of being involved in the intervention, or it may be that parents gain a higher awareness of the sibling’s needs due to the intervention. The increased time spent together between the sibling and parent could have an influence upon the result, but it is challenging to formally record this for it to be considered in the analysis (Houtzager et al. 2001; Williams et al. 2003).

Several of the studies that considered both physical and mental chronic health conditions gave justification as to why they chose to do so (Giallo and Gavidia-Payne 2008; Williams et al. 2003), but there is a lack of evidence as to which approach, disease-specific or broad, produces the optimum results. Evidence put forward by Vermaes et al. (2012) suggests that illnesses with a high morbidity and mortality may act as the largest moderating factors. Therefore, it may be beneficial to focus on siblings of children and young people that have a high impact and high mortality rate condition, regardless of whether the illness is categorised as physical or mental.

### Strengths and Limitations

Our study is the first to synthesise the current literature evaluating interventions offered to siblings of children with chronic physical or mental health conditions or both. Previous reviews have attempted to separate out the two groups, by physical or mental health conditions, and no previous meta-analysis has been completed in this area. The use of the broad approach allows a more complete picture of interventions currently available to siblings, by considering studies that have evaluated interventions focused on siblings of children and young people with a chronic mental or physical health condition together. It has also highlighted how it may not be the most advantageous approach to consider these groups separately. Consideration has been given to various forms of interventions and has highlighted the importance of more robust and replicable research in this area.

The heterogeneity of the studies included in the review makes it difficult to draw firm conclusions regarding the effectiveness of psychological interventions aimed at siblings of children with chronic conditions. The studies were typically subject to low sample sizes, poor methodology and short follow-up periods; less than a quarter (24%) of the included studies report on a follow-up beyond 1 month post-intervention. Although it is advantageous that this analysis has included studies that consider both physical and mental health conditions, this may also have increased the level of heterogeneity and reduced the clinical relevance, relative to reviews that have focused on solely mental or physical health conditions (Tudor and Lerner 2015). It should also be noted that the included studies were all from developed countries, and therefore, the results from this analysis cannot be generalised to those in low- and middle-income countries.

Not many studies were included in the meta-analysis due to a lack of consistent and compatible data. Of the included eight, five used an uncontrolled pre-post study design (Besier et al. 2010; Cebula 2012; Dolgin et al. 1997; Kryzak et al. 2015; Lobato and Kao 2002; Smith and Perry 2005) which makes causality difficult to establish. The four studies included in the meta-analysis that used a treatment–control design used various control groups: One used a waitlist control (Giallo and Gavidia-Payne 2008), one used the participants that refused to participate in the intervention (McLinden et al. 1991), another used a data sample from the general population (Besier et al. 2010), and finally, Cebula (2012) used a retrospective design, and thus, their control are willing subjects who have not previously/not currently using the intervention which limits the generalisability of the findings.

Further limitations of the included studies include a lack of acknowledgement for potential positive impacts of having a sibling with a chronic health condition. There is also a lack of consideration for the influence of parent-reported relative to child-reported outcomes. Cebula (2012) reported measures from parents, siblings and teachers to attempt to deal with this issue. In her analysis, she found that the child or young person reported themselves more negatively on two of the five domains of the SDQ, but they also appeared to have a slightly more positive perception of the sibling relationship, particularly empathy which may be due to the parents’ greater attention to negative interactions (Cebula 2012; Rivers and Stoneman 2003). How these change and influence results following intervention may be an important consideration.

Across all studies, there were 23 outcomes considered; sufficient data were reported to combine behaviour and knowledge scores in a meta-analysis. The results of this analysis were limited by the small sample sizes of previous studies, along with methodological problems due to the lack of consistency in measures being used, type and protocol of interventions, and time points being considered. It would be more beneficial if intervention studies that evaluated the same form of intervention, with the same type of population and using the same outcome measures could be statistically combined; unfortunately, with the current literature this is not possible.

### Directions for Future Research

The primary recommendation from this review and meta-analysis is the need for stronger evidence, such as RCTs, which also capture a larger more representative sample of the population. Which tools should be used in evaluations also requires deliberation to help encourage consistency across studies.

Furthermore, studies should be conducted that include siblings of children with both mental and physical health conditions. It may also be important to consider potential moderating factors, including protective factors, which could help target and tailor support services to those most in need. For instance, considering different populations based on moderating factors, i.e. high/low burden, using the same intervention protocol would provide more informative evaluations in this area of research.

## Conclusion

This review and meta-analysis improves upon the current literature by combining the existing findings in a systematic and robust manner, providing transparent results and reducing potential sources of bias. It is concluded that psychological well-being interventions for siblings of children and young people with chronic physical and mental health conditions lead to an improvement in illness knowledge and an improvement in externalising and internalising behaviour scores, when using a pre-post one group study design. The findings from the systematic review are mixed and inconsistent, which emphasises the need for additional work that better establishes the needs, appropriate methodologies and evaluation techniques for interventions offered to siblings of children with chronic health conditions.

## Electronic supplementary material

Below is the link to the electronic supplementary material.

Supplementary material 1 (DOCX 23 kb)

## Footnotes

1 OECD-32 average proportion of households with two or more children is reported as 54.6%, which is multiplied by our estimated prevalence estimates (13–32%) to achieve our estimate of children that have a sibling with a chronic health condition. “Children” in this instance are generally defined here as dependent resident children under 25 and include both biological children and step- or adopted children or any other children in the household, though exact definitions do vary across countries.

2 From the twelve studies which reported average age.

3 As determined through behavioural observations of sibling dyads.
