# Supplementary material for: Impact of Well-being Interventions for Siblings of Children and Young People with a Chronic Physical or Mental Health Condition: A Systematic Review and Meta-Analysis
Source: Clin Child Fam Psychol Rev. 2018 Feb 15;21(2):246–65. doi: 10.1007/s10567-018-0253-x (PMC5899110; doi:10.1007/s10567-018-0253-x)
Supplement: Supplementary file 1 — Supplementary material 1 (DOCX 23 kb) [file 10567_2018_253_MOESM1_ESM.docx]

## Online Resource 1: Full Search Strategy

| **Database** | **Topic/Headings/Subject/MeSH** | **Article Title, Abstract, Keywords** | **All Text/Key terms** | **Final Search** |
| --- | --- | --- | --- | --- |
| **PsycINFO** | 1. Siblings 2. Chronic disease 3. Mental Health 4. Quality of Life or Quality Adjusted Life Years 5. Social Support |  | 1. sibling* or brother* or sister* 2. (chronic or lifelong or recur* or incurable or ineradicable) AND (disease* or illness* or condition* or syndrome* or disorder*) 3. “mental health” or “psychological wellbeing” or stress* or anxiety* or depress* or psychosocial 4. “quality of life” or QoL or “quality adjusted life year” or QALY or “health related quality of life” or HRQoL 5. intervention* 6. (support or self-help) and (group* or peer* or mental-health or social or group* or school* or famil* or early) | 1. 1 or 6 2. 2 or 7 3. 3 or 8 4. 4 or 9 5. 14 or 15 6. 5 or 10 or 11 7. 12 and 13 and 16 and 17 |
| **PsycEXTRA** | 1. Siblings or Brothers or Sisters 2. Chronic illness 3. Mental health or Well Being 4. Quality of Life 5. Intervention or early intervention or family intervention or group intervention or school based intervention 6. Support Groups or mental health services or self-help techniques or social support |  | 1. sibling* or brother* or sister* 2. (chronic or lifelong or recur* or incurable or ineradicable) AND (disease* or illness* or condition* or syndrome* or disorder*) 3. mental health or psychological wellbeing or stress* or anxiety* or depress* or psychosocial 4. quality of life or QoL or quality adjusted life year or QALY or health related quality of life or HRQoL 5. intervention* 6. (support or self-help) and (group* or peer* or mental-health or social or group* or school* or famil* or early) | 1. 1 or 7 2. 2 or 8 3. 3 or 9 4. 4 or 10 5. 15 or 16 6. 5 or 6 or 11 or 12 7. 13 and 14 and 17 and 18 |
| **Embase** | 1. Sibling 2. Chronic disease 3. Mental health or psychological well being 4. Quality of life or quality adjusted life year 5. Intervention study or early intervention or early childhood intervention 6. Social support or support group |  | 1. sibling* or brother* or sister* 2. (chronic or lifelong or recur* or incurable or ineradicable) AND (disease* or illness* or condition* or syndrome* or disorder*) 3. mental health or psychological wellbeing or stress* or anxiety* or depress* or psychosocial 4. quality of life or QoL or quality adjusted life year or QALY or health related quality of life or HRQoL 5. intervention* 6. (support or self-help) and (group* or peer* or mental-health or social or group* or school* or famil* or early) | 1. 1 or 7 2. 2 or 8 3. 3 or 9 4. 4 or 10 5. 15 or 16 6. 5 or 6 or 11 or 12 7. 13 and 14 and 17 and 18 |
| **PubMed** | 1. Siblings 2. Chronic disease 3. mental health 4. quality of life or quality adjusted life years 5. social support |  | 1. sibling* or brother* or sister* 2. (chronic or lifelong or recur* or incurable or ineradicable) AND (disease* or illness* or condition* or syndrome* or disorder*) 3. “mental health” or “psychological wellbeing” or stress* or anxiety* or depress* or psychosocial 4. “quality of life” or QoL or “quality adjusted life year” or QALY or “health related quality of life” or HRQoL 5. intervention* 6. (support or self-help) and (group* or peer* or mental-health or social or group* or school* or famil* or early) | 1. 1 or 6 2. 2 or 7 3. 3 or 8 4. 4 or 9 5. 14 or 15 6. 5 or 10 or 11 7. 12 and 13 and 16 and 17 |
| **CINAHL** | 1. Siblings or brothers or sisters 2. chronic disease or chronic illness or long term conditions or chronic conditions 3. mental health or well-being 4. quality of life or health related quality of life 5. intervention or early intervention or family intervention or group intervention or school based intervention 6. support groups or mental health services or self-help techniques or social support |  | 1. sibling* or brother* or sister* 2. (chronic or lifelong or recur* or incurable or ineradicable) AND (disease* or illness* or condition* or syndrome* or disorder*) 3. mental health or psychological wellbeing or stress* or anxiety* or depress* or psychosocial 4. quality of life or QoL or quality adjusted life year or QALY or health related quality of life or HRQoL 5. intervention* 6. (support or self-help) and (group* or peer* or mental-health or social or group* or school* or famil* or early) | 1. 1 or 7 2. 2 or 8 3. 3 or 9 4. 4 or 10 5. 15 or 16 6. 5 or 6 or 11 or 12 7. 13 and 14 and 17 and 18 |
| **Scopus** |  | 1. sibling* or brother* or sister* 2. (chronic or lifelong or recur* or incurable or ineradicable) AND (disease* or illness* or condition* or syndrome* or disorder*) 3. (“mental health” or “psychological wellbeing” or stress* or anxiety* or depress* or psychosocial) OR (“quality of life” or QoL or “quality adjusted life year” or QALY or “health related quality of life” or HRQoL) 4. intervention* or ((support or self-help) and (group* or peer* or mental-health or social or group* or school* or famil* or early)) |  | 1 and 2 and 3 and 4 |
| **Web of Science** | 1. sibling* or brother* or sister* 2. (chronic or lifelong or recur* or incurable or ineradicable) AND (disease* or illness* or condition* or syndrome* or disorder*) 3. (“mental health” or “psychological wellbeing” or stress* or anxiety* or depress* or psychosocial) OR (“quality of life” or QoL or “quality adjusted life year” or QALY or “health related quality of life” or HRQoL) 4. Intervention* or ((support or self-help) and (group* or peer* or mental-health or social or group* or school* or famil* or early)) |  |  | 1 and 2 and 3 and 4 |

## Online Resource 2: Quality Assessment Results

| Author | Year | Selection Bias | Study Design | Confounders | Blinding | Data Collection Methods | Withdrawals and Drop-Outs | Overall |
| --- | --- | --- | --- | --- | --- | --- | --- | --- |
| Besier et al. | 2010 | ✶✶ | ✶✶ | ✶ | ✶✶ | ✶✶✶ | ✶ | ✶ |
| Cebula | 2012 | ✶ | ✶✶ | ✶✶✶ | ✶✶ | ✶✶✶ | N/A | ✶✶ |
| D'Arcy et al. | 2005 | ✶✶ | ✶✶ | ✶✶✶ | ✶✶ | ✶ | ✶ | ✶ |
| Dolgin et al. | 1997 | ✶✶ | ✶✶ | ✶✶✶ | ✶✶ | ✶ | ✶✶✶ | ✶✶ |
| Evans, Jones & Mansell | 2001 | ✶ | ✶✶ | ✶✶✶ | ✶✶ | ✶ | ✶ | ✶ |
| Giallo & Gavidia-Payne | 2008 | ✶ | ✶✶✶ | ✶✶✶ | ✶✶ | ✶ | ✶✶ | ✶ |
| Granat et al. | 2012 | ✶✶ | ✶✶ | ✶✶✶ | ✶✶ | ✶ | ✶✶✶ | ✶✶ |
| Heiney et al. | 1990 | ✶✶ | ✶✶ | ✶ | ✶✶ | ✶ | ✶ | ✶ |
| Houtzager, Frootenhuis, & Last | 2001 | ✶ | ✶✶ | ✶✶✶ | ✶✶ | ✶ | ✶✶ | ✶ |
| Kiernan et al. | 2004 | ✶ | ✶✶ | ✶✶✶ | ✶✶ | ✶ | ✶ | ✶ |
| Kryzak et al. | 2014 | ✶ | ✶✶ | ✶✶✶ | ✶✶ | ✶✶✶ | ✶✶ | ✶✶ |
| Lobato & Kao | 2002 | ✶ | ✶✶ | ✶✶✶ | ✶✶ | ✶✶✶ | ✶✶✶ | ✶✶ |
| McLinden et al. | 1991 | ✶ | ✶✶ | ✶ | ✶✶ | ✶ | ✶✶ | ✶ |
| Phillips | 1999 | ✶ | ✶✶✶ | ✶✶✶ | ✶✶ | ✶✶✶ | ✶✶✶ | ✶✶ |
| Sidhu et al. | 2006 | ✶✶ | ✶✶ | ✶✶✶ | ✶✶ | ✶✶✶ | ✶✶✶ | ✶✶✶ |
| Smith & Perry | 2005 | ✶✶ | ✶✶ | ✶✶✶ | ✶✶ | ✶✶✶ | ✶✶✶ | ✶✶✶ |
| Williams et al. | 2003 | ✶ | ✶✶✶ | ✶✶✶ | ✶✶ | ✶✶✶ | ✶✶✶ | ✶✶ |
| ✶✶✶= Strong; ✶✶= Moderate; ✶ = Weak | | | | | | | | |
